# Identification of IL10RA by Weighted Correlation Network Analysis and *in vitro* Validation of Its Association With Prognosis of Metastatic Melanoma

**DOI:** 10.3389/fcell.2020.630790

**Published:** 2021-01-08

**Authors:** Si Cheng, Zhe Li, Wenhao Zhang, Zhiqiang Sun, Zhigang Fan, Judong Luo, Hui Liu

**Affiliations:** ^1^Department of Radiotherapy, The Affiliated Changzhou No. 2 People's Hospital of Nanjing Medical University, Changzhou, China; ^2^Department of Dermatology, Graduate School of Dalian Medical University, Dalian, China; ^3^Department of Breast Surgery, Shuguang Hospital Affiliated to Shanghai University of Traditional Chinese Medicine, Shanghai, China; ^4^Aliyun School of Big Data, Changzhou University, Changzhou, China; ^5^Department of Oncology, Affiliated 3201 Hospital of Xi'an Jiaotong University, Hanzhong, China

**Keywords:** skin cutaneous melanoma (SKCM), metastatic melanoma, WGCNA, IL10RA, survival analysis, prognostic biomarker

## Abstract

Skin cutaneous melanoma (SKCM) is the major cause of death for skin cancer patients, its high metastasis often leads to poor prognosis of patients with malignant melanoma. However, the molecular mechanisms underlying metastatic melanoma remain to be elucidated. In this study we aim to identify and validate prognostic biomarkers associated with metastatic melanoma. We first construct a co-expression network using large-scale public gene expression profiles from GEO, from which candidate genes are screened out using weighted gene co-expression network analysis (WGCNA). A total of eight modules are established via the average linkage hierarchical clustering, and 111 hub genes are identified from the clinically significant modules. Next, two other datasets from GEO and TCGA are used for further screening of biomarker genes related to prognosis of metastatic melanoma, and identified 11 key genes via survival analysis. We find that IL10RA has the highest correlation with clinically important modules among all identified biomarker genes. Further *in vitro* biochemical experiments, including CCK8 assays, wound-healing assays and transwell assays, have verified that IL10RA can significantly inhibit the proliferation, migration and invasion of melanoma cells. Furthermore, gene set enrichment analysis shows that PI3K-AKT signaling pathway is significantly enriched in metastatic melanoma with highly expressed IL10RA, indicating that IL10RA mediates in metastatic melanoma via PI3K-AKT pathway.

## 1. Introduction

In the past few decades, the incidence of skin cutaneous melanoma (SKCM) has gone up faster than any other solid tumors (Eggermont et al., 2014). In 2018, the estimated number of new cases of SKCM was 287,723, among which 60,712 died (Bray et al., 2018). Widespread metastases to the skin, subcutaneous, lymphatic system, lung and other non-pulmonary visceral are implicated in poor patient survival, which is the major obstacle to improve prognosis (Balch et al., 2009). The median survival time with metastatic melanoma is only 6–9 months and the 5-years survival rate is under 5% with traditional therapies (Agarwala, 2009). Therefore, the comprehensive understanding of melanoma progression and the molecular mechanism underlying the growth and metastasis of melanoma are of great significance.

Biomarkers for diagnosis and prognosis of cutaneous melanoma have drawn intensive attentions from academic and industrial communities in recent years, and many studies have identified plenty of effective biomarkers and therapeutic targets in metastatic melanoma. For example, increasing concentrations of serum S100 calcium-binding protein B (S100B), lactate dehydrogenase (LDH), melanoma-inhibiting activity (MIA), YKL40 and tumor-associated antigen 90 immune complex (TA90IC) are all strongly associated with overall survival, suggesting their function as prognostic factor in late-stage malignant melanoma (Gogas et al., 2009). Linked by a network of overlapping functions in melanoma progression, melanoma cell adhesion molecule (MCAM), galectin-3 (Gal-3), chondroitin sulfate proteoglycan 4 (CSPG4), matrix metalloproteinase 2 (MMP-2), and paired box 3 (PAX-3) potentially act as biomarkers and targets in melanoma metastasis (Dye et al., 2013). Hofmann et al. (2011) found the serum MIA level was closely related to the status of lymph nodes in the affected basin of stage III melanoma patients. However, the underlying mechanism of metastatic melanoma is still unclear.

The high-throughput sequencing technique has open a new door to the study of clinical outcomes and pathological mechanism of various cancers. However, traditional differential expression analysis is not efficient to uncover the interconnection among genes with similar biological functions. Weighted gene co-expression network analysis (WGCNA) has been proposed to address high-dimensional data, especially for the free-scale gene coexpression networks based on the likelihood of expression profile between genes (Tang et al., 2018). A set of genes that are highly correlated will be grouped into a module and the module could be associated with different clinical traits (Giulietti et al., 2016). WGCNA (Langfelder and Horvath, 2008) has been successfully applied to evaluate the associations between gene modules and clinical features. For example, Wang et al. (2020) used WGCNA to identify human T cell lymphotropic virus-1 (HTLV-1) infection and mTOR signaling pathway, as well as the AKT1 and MAPK14 genes that might serve as biomarkers and targets for precise diagnosis and treatment of ischemic stroke. In addition, hub genes can be identified based on the correlation between the genes and the module eigengenes (MEs).

In this work, we conducted WGCNA on the GEO dataset GSE22153 to identify prognostic biomarker genes of cutaneous melanoma. There are eight gene modules established via the average linkage hierarchical clustering and 111 candidate genes identified in the clinically significant modules. Subsequently, two other RNA-seq datasets together with clinical information of metastatic melanoma from GEO and TCGA datasets were used for further screening for hub genes related to prognosis. As a result, 11 hub genes that are significantly correlated with prognosis in both datasets were identified via survival analysis. Especially, the interleukin 10 receptor subunit alpha (IL10RA) shows the highest correlation with clinically important modules. We have conducted further *in-vitro* biochemical experiments, including CCK8 assays, wound-healing assays and transwell assays, and verified that IL10RA can significantly inhibit the proliferation, migration and invasion of melanoma cells. Furthermore, gene set enrichment analysis revealed that PI3K-AKT signaling pathway is significantly enriched in metastatic melanoma with highly expressed IL10RA, indicating its significance as potential biomarkers.

## 2. Materials and Methods

### 2.1. Data Resource and Pre-processing

The gene expression profiles are downloaded from the Gene Expression Omnibus (GEO) database (Edgar et al., 2002) and The Cancer Genome Atlas (TCGA) database (Tomczak et al., 2015). From GEO, we retrieve two datasets GSE22153 and GSE22154, which includes 57 metastatic melanoma samples associated with four molecular subtypes (high-immune response, pigmentation, normal-like and proliferative) and 22 metastatic melanoma samples with clinical information, respectively. Also, we get 367 metastatic melanoma samples with clinical information from TCGA. The GSE22153 dataset is used to run WGCNA to identify candidate hub genes, while the GEO GSE22154 and TCGA data are used to perform survival analysis for further screening of prognostic genes.

### 2.2. Construction of Gene Coexpression Network

We construct a gene co-expression network using the WGCNA package (Horvath and Dong, 2008; Mason et al., 2009). First, we calculate the Pearson correlation coefficient (PCC) for all paired genes. Second, an adjacency matrix is constructed using a power function as below 1:

(1) $$\begin{array}{r} A_{ij}=|p_{ij}{|}^{\beta } \end{array}$$

in which *A*_*ij*_ is the adjacency element between gene *i* and gene *j*, *p*_*ij*_ represents the PCC between gene *i* and gene *j*, and β is a soft thresholding parameter that could stress strong correlations between genes and penalize weak correlations. In this study, the power of β is set to 4 (scale free *R*^2^ = 0.9) to ensure a scale-free network.

Next, the adjacency matrix is transformed into topological overlap matrix (TOM) so that the genes with similar expression profiles are clustered into modules using the average-linkage hierarchical clustering method. Of note, the minimum base number of each gene network module is set to 30 in this study. As shown in Equation (2) (Langfelder and Horvath, 2008), the element of TOM can be calculated as

(2) $$\begin{array}{r} TOM_{ij}=\frac{\sum_{k=1}^{N}A_{ik}\cdot A_{kj}+A_{ij}}{min\left(C_{i},C_{j}\right)+1-A_{ij}} \end{array}$$

in which *A*_*ij*_ refers to the beta power of the correlation coefficient between gene *i* and gene *j*, *A*_*ik*_ and *A*_*kj*_ are similar variables. The numerator of this formula refers to the sum of indirect correlation and direct correlation between gene *i* and gene *j*. $C_{i}=\underset{k}{\sum }A_{ik}$ and $C_{j}=\underset{k}{\sum }A_{jk}$ means the connectivity of gene *i* and gene *j*, respectively.

### 2.3. Discovery of Important Network Modules

The correlation between modules and clinical subtypes is calculated according to the feature vector of each network module. Module eigengenes actually formulate the expression patterns of the all genes within a given module into a single characteristic expression profile. Module eigengenes can be regarded as the first principal component of the gene module. The correlation between each gene in these modules and each subtype (high-immune response, pigmentation, normal-like and proliferative) is quantified by gene significance (GS) value. Accordingly, module significance (MS) of a certain module is defined as the averaged GS values of all genes included in it. Modules are ranked according to the MS score, and the top two modules are considered as key modules relevant to clinical outcomes for further analysis.

### 2.4. Identification of Candidate Biomarkers

Hub genes in the coexpression network are a class of genes that have high connectivity within a network module and significantly correlated with biological function (Chen et al., 2017). In this study, the connectivity of genes is measured by absolute value of the module membership (MM) score, which represents the PCC between certain gene and module eigengene. Besides, we measure the absolute value of gene significance (GS) score, which represents the correlation between the genes in these modules and each type of phenotype (Yang et al., 2018). We screen candidate genes using the cut-off criteria |*MM*|≥0.8 and |*GS*|≥0.2, because such genes are biologically meaningful. The |*MM*|≥0.8 indicates that the gene is strongly related to module, and |*GS*|≥0.2 requires that the gene expression profile is also closely related to phenotpical subtype. Finally, the degree of connectivity of each gene is the sum of the edge attributes of the genes connected to it. The higher the connectivity, the stronger the biological function of the gene. We select the top 30 hub genes to cover most genes that reach the MM and GS thresholds in each module.

### 2.5. Co-expression Network Analysis and Functional Enrichment Analysis

Based on the protein-protein interactions (PPIs) derived from STRING (Szklarczyk et al., 2016), we construct PPI networks of candidate biomarker genes in each clinically significant module. The PPI networks are visualized using Cytoscape. Besides, Gene Ontology (GO) enrichment analysis and Kyoto Encyclopedia of Genes and Genomes (KEGG) pathway analysis are performed using the R package clusterProfiler (Yu et al., 2012).

### 2.6. Determination of Biomarker Genes Significant to Survival

Based on the GEO GSE22154 and TCGA datasets, Kaplan-Meier survival analysis (Györffy et al., 2010) is performed to explore the relationship between the expression level of candidate biomarker genes and the survival days of metastatic melanoma patients (Lánczky et al., 2016). The GEO GSE22154 and TCGA datasets include 22 and 367 metastatic melanoma samples, respectively. Because they come from different data source, we run survival analysis independently on these two datasets, as the sample sizes of both datasets are enough for statistical significance. The log rank *p* values and the hazard ratio are calculated. The genes with statistical significance (*p* < 0.05) to prognosis in both datasets are determined as the biomarkers related to survival, on which further *in-vitro* biochemical experiments are conducted.

### 2.7. *In-vitro* Biochemical Validation of Biomarker Gene

Human malignant melanoma cell line (A375) and mouse skin melanoma cell line (B16-F10) are purchased from Chinese Academy of Sciences Cell Bank (Shanghai, China). Cell proliferation is measured using cell counting kit-8 (CCK-8). A375 and B16-F10 cells are transfected with si-IL10RA or si-Control in 6-well plates. After 24 h, the cells are seeded into 96-well plates and cultured for 24, 48, 72, and 96 h, respectively. The absorbance at 450 nm is measured to determine the cell viability. Cell migration ability is measured using wound-healing assays, where cells seed in a 6-well plate. Until the cell confluence reaches 95%, a scratch wound is generated using a sterile 200 μL pipette tip. Next, the scratches are photographed at 0 h and 24 h. Cell invasion ability is measured by transwell assays using the transwell chambers, which are pre-coated with Matrigel. The lower chamber is added to DMEM with 20% FBS, and melanoma cells in serum-free medium are placed in the upper chamber. The cells invaded into the lower chamber is fixed in 4% paraformaldehyde after incubation for 24 h and stained with crystal violet.

### 2.8. Gene Set Enrichment Analysis

A total of 367 metastatic melanoma samples in TCGA are divided into high-expression and low-expression groups according to the median expression levels of hub gene. To study the potential mechanisms of metastatic melanoma, GSEA between the two groups is performed using the Java GSEA implementation (Subramanian et al., 2005), where *FDR* < 0.05 is set as the cut-off criteria.

## 3. Results

### 3.1. Parameter Optimization and Determination of Network Modules

**Figure 1:**
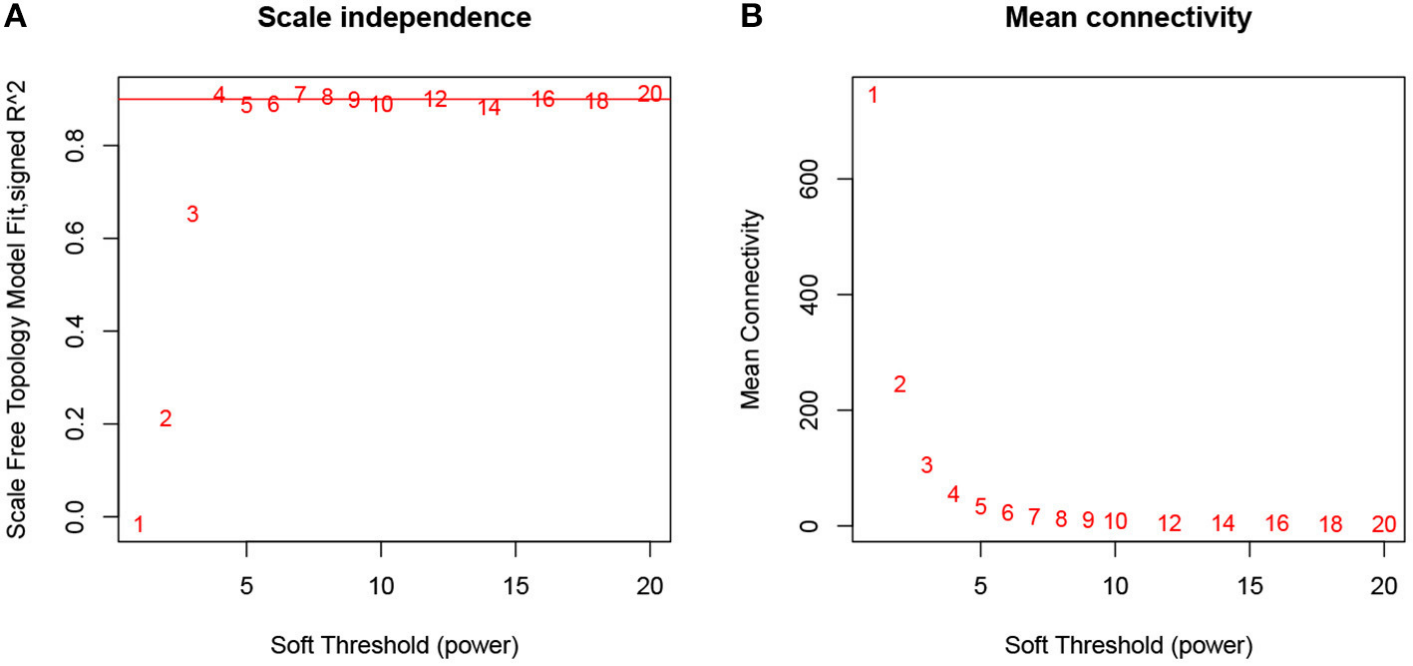
Determination of soft-thresholding power in WGCNA analysis. **(A)** Analysis of the scale-free fit index for various soft-thresholding powers β. **(B)** Analysis of the mean connectivity for various soft-thresholding powers.

The expression profiles of 57 samples covering four molecular subtypes were included in the coexpression analysis. We processed 12,633 gene expression profiles using variance analysis on the GSE22153 dataset. The top quartile threshold (top 25%), a frequently used selection criterion, is used to select most variant genes. As a result, 3,658 unique genes with highest variances were screened out for further WGCNA analysis. To ensure that the network was a scale-free network, we ran empirical analysis to choose an optimal parameter β. As shown in Figure 1, both the scale-free topology model fit index (R^2^) and mean connectivity reach steady status when β is equal to 4.

**Figure 2:**
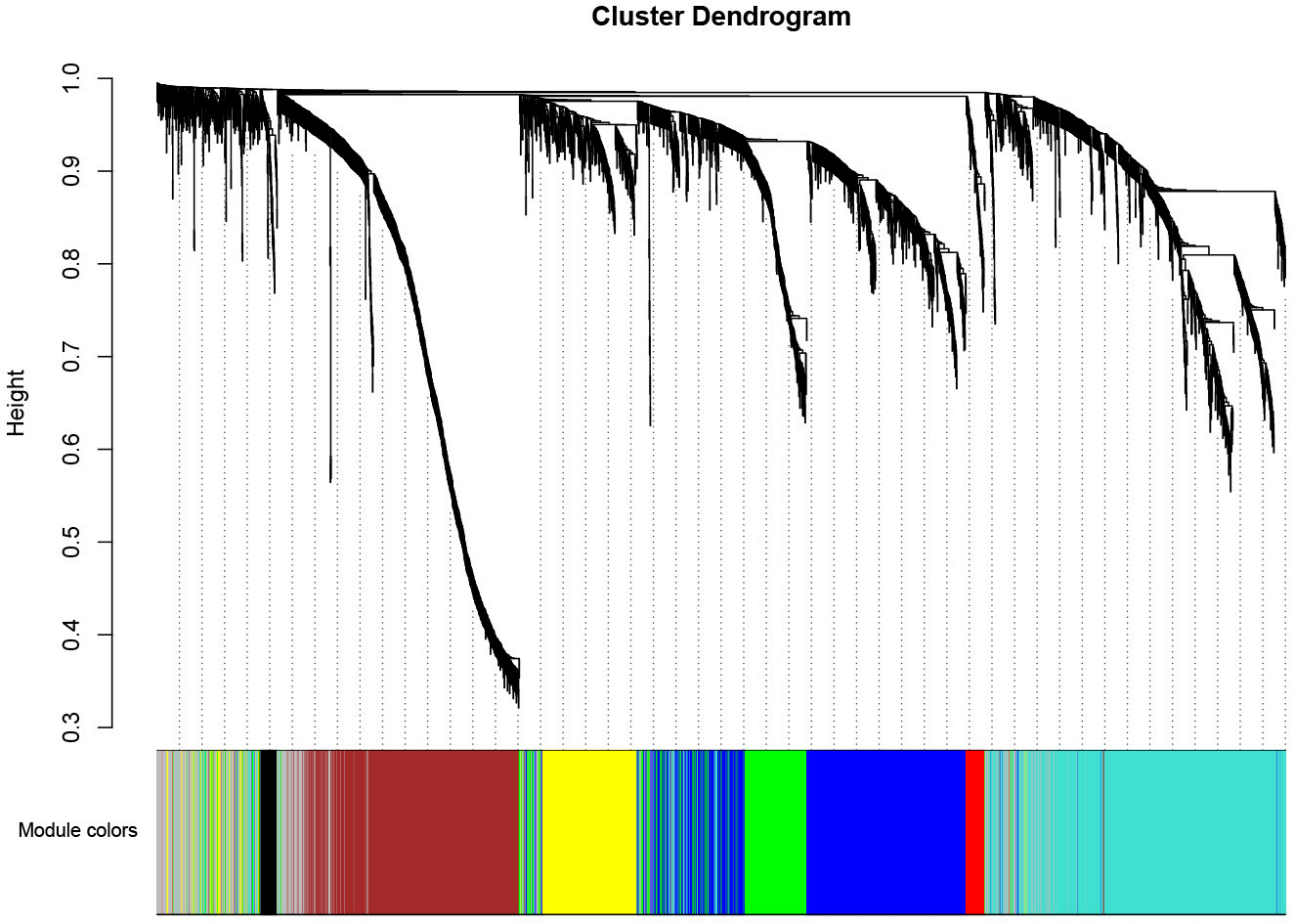
Dendrogram of all differentially expressed genes clustered based on a dissimilarity measure (1-TOM).

**Figure 3:**
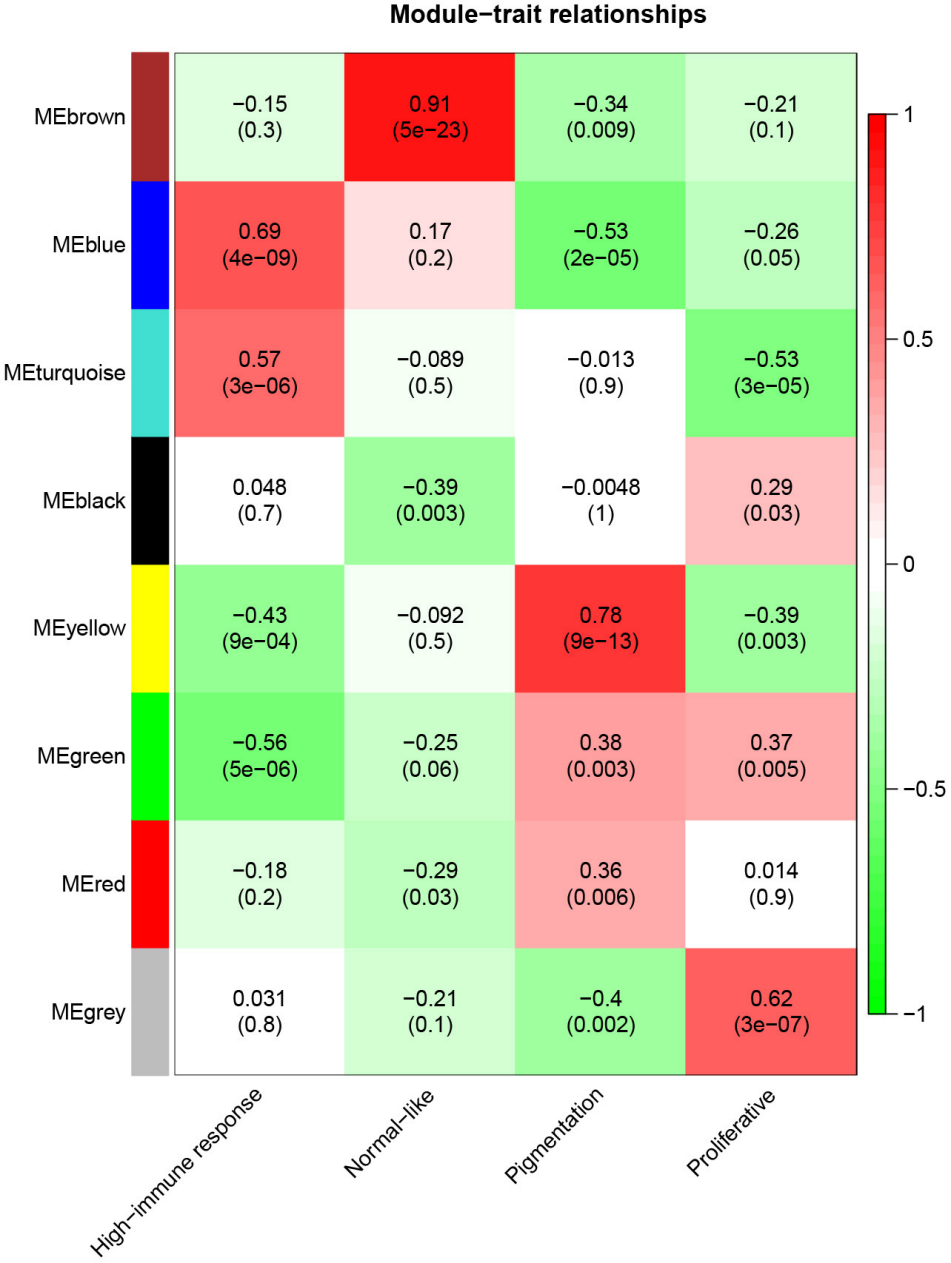
Heatmap of the correlation between module eigengenes and clinical traits of metastatic melanoma.

A total of eight modules were identified via average linkage hierarchical clustering and each module is represented in different color, as illustrated in Figure 2. To explore the correlation between module eigengenes and clinical traits, we plot a heat map shown in Figure 3. Each column in Figure 3 displayed the correlation and corresponding *p*-value. Red color represents positive correlations and green color represents negative ones. The darker the color, the stronger the correlation coefficient. We found that each clinical subtype is closely related to certain module, namely, the expression profiles of a set of genes significantly characterize each subtype. For example, we can find from Figure 3 that the high-immune response subtype is most relevant and positively correlated with the blue module. Its correlation coefficient is 0.69 and the *p*-value is 3e-06. Furthermore, the specific GS value across the modules in each subtype is shown in Supplementary Figure 1, we found that four module eigengenes, colored in blue, yellow, brown, and turquoise, have the highest correlation with the subtypes high-immune response, pigmentation, normal-like and proliferative, respectively. Accordingly, they were selected as the clinically significant modules for further analysis.

### 3.2. Candidate Genes Significantly Associated With Metastatic Melanoma

**Figure 4:**
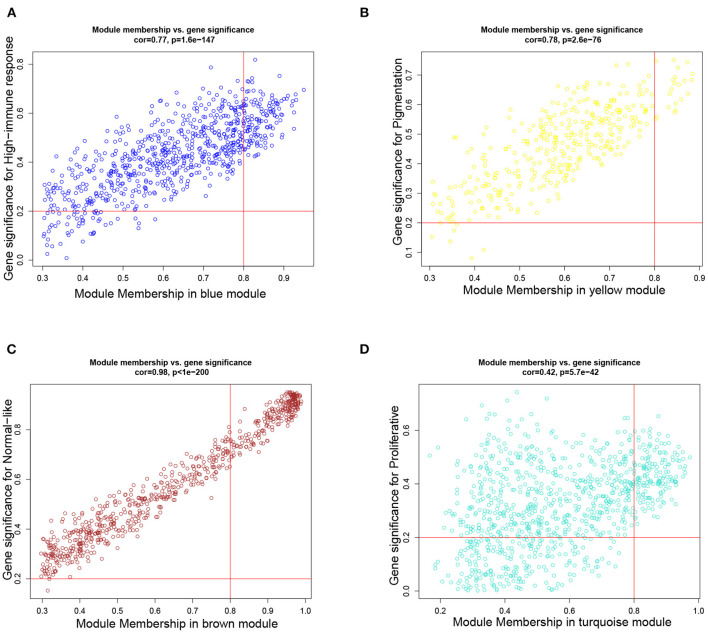
Scatter plots of the degree and *P*-value of Cox regression in dataset. The x-axis indicates the degree of regression, the y-axis indicates the gene significance. Each circle represents a gene. **(A)** A scatterplot of gene significance for highimmune response versus module membership in the blue module. **(B)** A scatterplot of gene significance for pigmentation versus module membership in the yellow module. **(C)** A scatterplot of gene significance for normal-like versus module membership in the brown module. **(D)** A scatterplot of gene significance for proliferative versus module membership in the turquoise module.

**Figure 5:**
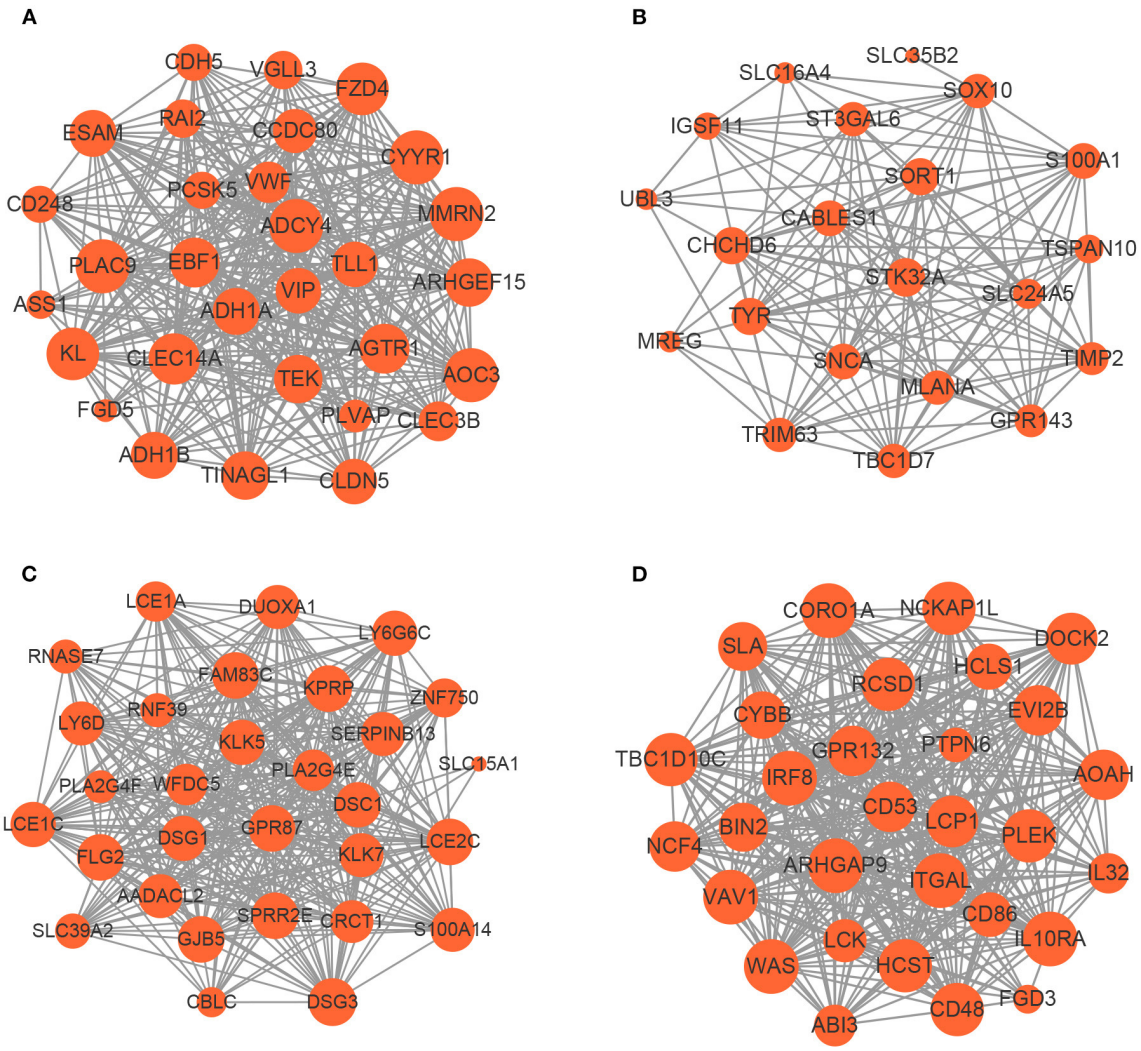
The network of hub genes in the **(A)** blue module, **(B)** yellow module, **(C)** brown module, and **(D)** turquoise module. Nodes represent genes and node size indicates weighted degree score.

Based on the aforementioned cut-off criteria |*MM*|≥0.8 and |*GS*|≥0.2, we subsequently sorted the genes according to their connectivity to select candidate biomarker genes, as shown in Figure 4. Note that only 21 genes are screened out in the yellow module, and then all of them are selected as candidate genes. For other three modules, top 30 genes that have high functional significance in these clinical modules are selected. In total, we obtained 111 candidate genes. Based on the PPI interactions derived from STRING, a PPI network covering these candidate genes in each module was constructed by Cytoscape, as shown in Figure 5.

To uncover the role of candidate genes in the pathogenesis of melanoma, GO and KEGG enrichment analysis were carried out. The GO analysis results revealed that the candidate biomarker genes in the biological process group were mainly enriched in epidermis development, neutrophil degranulation and keratinization. The molecular function group were mainly enriched in phosphotyrosine residue binding and Rho guanyl-nucleotide exchange factor activity. The genes in the cellular component group were significantly enriched in cell-cell junction and tertiary granule. Furthermore, our KEGG analysis demonstrated that candidate genes were mainly enriched in leukocyte transendothelial migration and tyrosine metabolism (see Supplementary Figure 2 for more detail). Our pathway enrichment analysis results showed that candidate genes are mainly related to the growth and structure of the skin. The malfunction of these genes may result in melanoma metastasis, as reported that Rho guanine-nucleotide exchange factor is necessary for effective melanoma metastasis (Lindsay et al., 2011).

### 3.3. Biomarkers Are Predictive of Prognosis of Metastatic Melanoma

**Figure 6:**
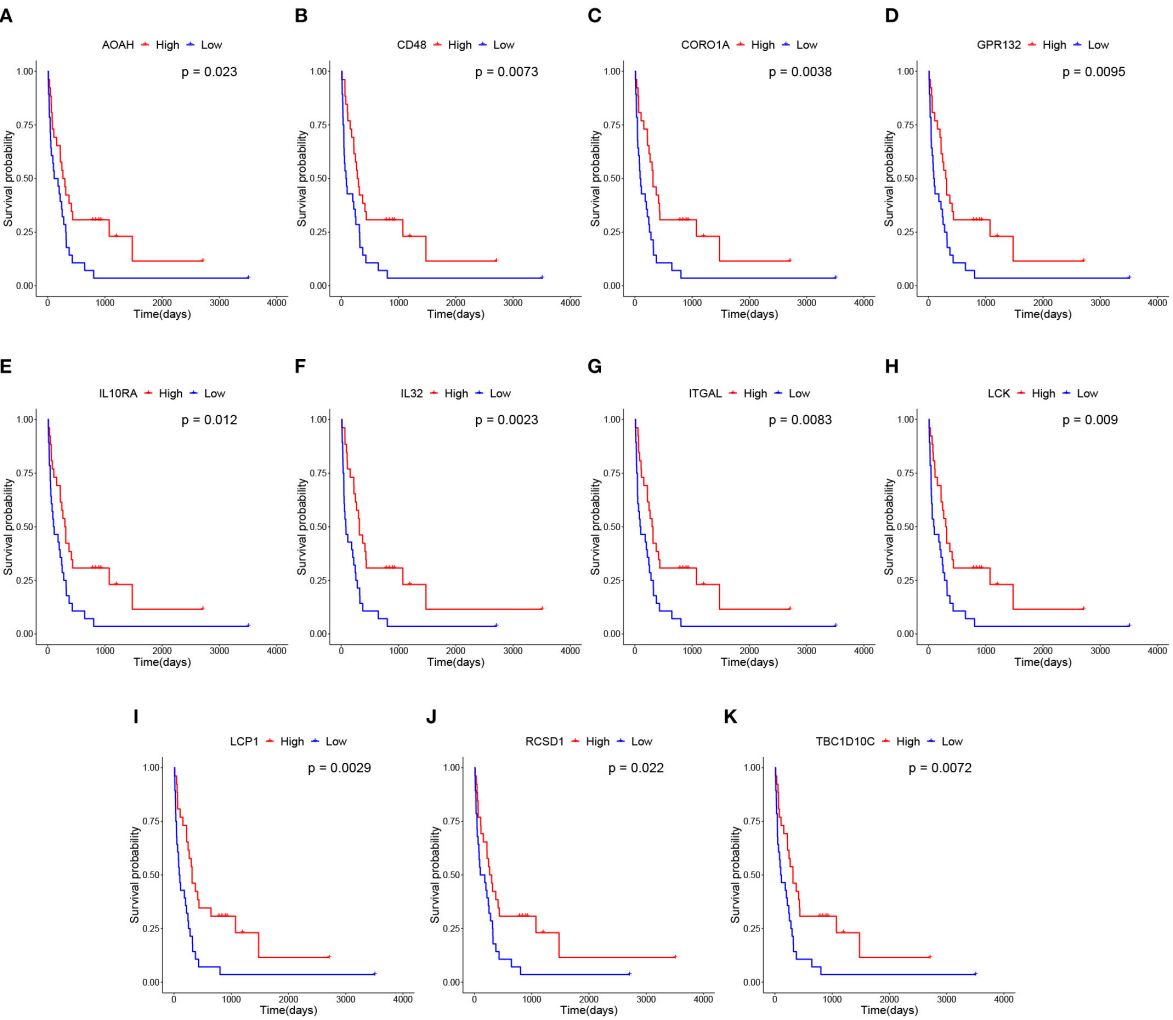
Survival analysis of 11 hub genes in metastatic melanoma in GEO GSE22154 dataset. Note that the red curves represent the samples with a highly expressed gene and the blue curves represent samples with a lowly expressed gene. **(A)** Overall survival (OS) of AOAH. **(B)** OS of CD48. **(C)** OS of CORO1A. **(D)** OS of GPR132. **(E)** OS of IL10RA. **(F)** OS of IL32. **(G)** OS of ITGAL. **(H)** OS of LCK. **(I)** OS of LCP1. **(J)** OS of RCSD1. **(K)** OS of TBC1D10C.

We subsequently conducted overall survival analysis on other two independent datasets from GEO and TCGA. The patients are stratified into high-level and low-level groups according to the median expression of the 111 candidate genes to further screen the prognostic genes. As shown in Figure 6, the expression profiles of 11 biomarker genes, including IL10RA, AOAH, CD48, IL32, CORO1A, GPR132, ITGAL, LCK, LCP1, RCSD1, and TBC1D10C, are significantly associated with overall survival of patients with metastatic melanoma on GEO GSE22154 dataset. The survival analysis results on TCGA dataset is shown in Supplementary Figure 3. Among these genes, several have been reported to be associated with the prognosis of SKCM. For example, the expression of IL-32γ and IL-32β is associated with increased cancer cell death in melanoma (Sloot et al., 2018). It has been reported that high expression of GPR132 resulted in cell cycle arrest and reduced oncogene transformation potential (Klatt et al., 2020). A few other studies also demonstrated that cutaneous melanomas from the immune transcriptomic subgroup that correlates with pathological lymphocytic infiltration also express elevated levels of LCK protein, which are associated with improved patient survival (Akbani et al., 2015). Niethammer et al. (2001) found that the obviously up-regulation in expression of CD48 and B7.1 on antigen-presenting cells provided further evidence for the effectiveness of the targeted Interleukin 2 therapy enhancing the immune response induced by an autologous oral DNA vaccine against murine melanoma. In addition, some of biomarker genes have been reported to be related to tumor development and tumor microenvironment. For example, Passon et al. found that in papillary thyroid carcinoma frequent deletion variants were detected in the 6q25.2 region containing the OPMR1 and IPCEF1 genes, and the 7q14.2 region containing the AOAH and ELMO1 genes. Besides, according to the risk classification of the American Joint Committee on Cancer stage and American Thyroid Association (ATA), deletion variants are more frequent in lower risk sample (Passon et al., 2015). Also, CORO1A is another important new member in integrin biology and plays key function in trafficking of polymorphonuclear neutrophils (PMNs) during innate immunity (Pick et al., 2017). The down regulation of ABL2, ITGAL, and SEMA4D in patients with increased mortality is indicative of a down-regulation of innate and acquired immunity (Ross et al., 2012). In fact, it has been reported that RCSD1-ABL1-positive B lymphoblastic leukemia is sensitive to tyrosine kinase inhibitors (Frech et al., 2017). The overexpression of LCP1 is correlated with increased infiltrating levels of CD8^+^ T cells, CD4^+^ T cells, macrophages, neutrophils and dendritic cells (Wang et al., 2008). TBC1D10C inhibits the Ras/MAPK signaling pathway and is a negative feedback inhibitor of the calcineurin signaling pathway (Pan et al., 2007). Overall, these genes play an important role in tumorigenesis, and we further conducted *in vitro* experiments to verify that the role of IL10RA gene in the prognosis of metastatic melanoma.

### 3.4. IL10RA Low Expression Associated to Poor Prognosis of Multiple Cancers

The module membership scores of the 11 candidate genes are listed in Table 1, from which we find that IL10RA achieves the highest score and thus selected as a biomarker gene for subsequent validation. The IL10RA gene encodes a protein that is a subunit of the interleukin-10 receptor. This receptor is reported to promote survival of progenitor myeloid cells through the insulin receptor substrate-2/PI 3-kinase/AKT pathway (Zhou et al., 2001).

**Table 1:** The module membership scores of 11 screened biomarker genes.

**Gene**	**Module membership score**	**Description**
IL10RA	0.971951725	Interleukin 10 receptor subunit alpha
CORO1A	0.954216393	Coronin 1A
ITGAL	0.950900031	Integrin subunit alpha L
LCP1	0.938553955	Lymphocyte cytosolic protein 1
RCSD1	0.93327131	RCSD domain containing 1
IL32	0.932559944	Interleukin 32
GPR132	0.925335767	G protein-coupled receptor 132
TBC1D10C	0.924839442	TBC1 domain family member 10C
CD48	0.919333672	CD48 molecule
AOAH	0.905119134	Acyloxyacyl hydrolase
LCK	0.874869931	LCK proto-oncogene, Src family tyrosine kinase

In fact, previous studies have reported the differential expression of IL10RA in tumors and non-tumor diseases. For example, IL10 deficient patients develop severe infantile-onset inflammatory bowel disease (IBD) (Shouval et al., 2017). Expression of IL10RA within the leukocyte population (CD45^+^ cells) was obviously lower in metastases than in both gliomas and meningiomas (Zadka et al., 2017). Li et al. found that macrophages but not Th17 cells expressed IL10RA on the cell surface, and lung Th17 cells respond poorly to IL10 stimulation but macrophages switch phenotype in response to IL10 stimulation. They also demonstrated that IL10-treated macrophages inhibited CD4^+^ T cell IL17 production and further suppresses lung tumorigenesis (Li et al., 2018). Other studies also demonstrated that programmed death-1 high tumor antigen (TA)-specific CD8^+^ T cells present in metastatic melanoma upregulate IL10R. IL10 acts directly on IL10R^+^ TA-specific CD8^+^ T cells to impede their expansion. IL-10 blockade adds to PD-1 blockade to further strengthen the expansion and functions of TA-specific CD8^+^ T cells, because TA-specific CD8^+^ T cells up-regulate IL10R upon PD-1 blockade (Sun et al., 2015). Moreover, it has been reported that the IL10RA Ser138Gly variant showed a weak association with the risk of all lymphoma combined (odds ratio [OR], 0.81; 95% CI, 0.65–1.02), mainly driven by the 50% risk reduction for Hodgkin's lymphoma (HL) (Nieters et al., 2006). These results indicate that IL10RA is an important biomarker for the prognosis of multiple type of cancers.

### 3.5. IL10RA Inhibits the Proliferation, Migration, and Invasion of Melanoma Cells

**Figure 7:**
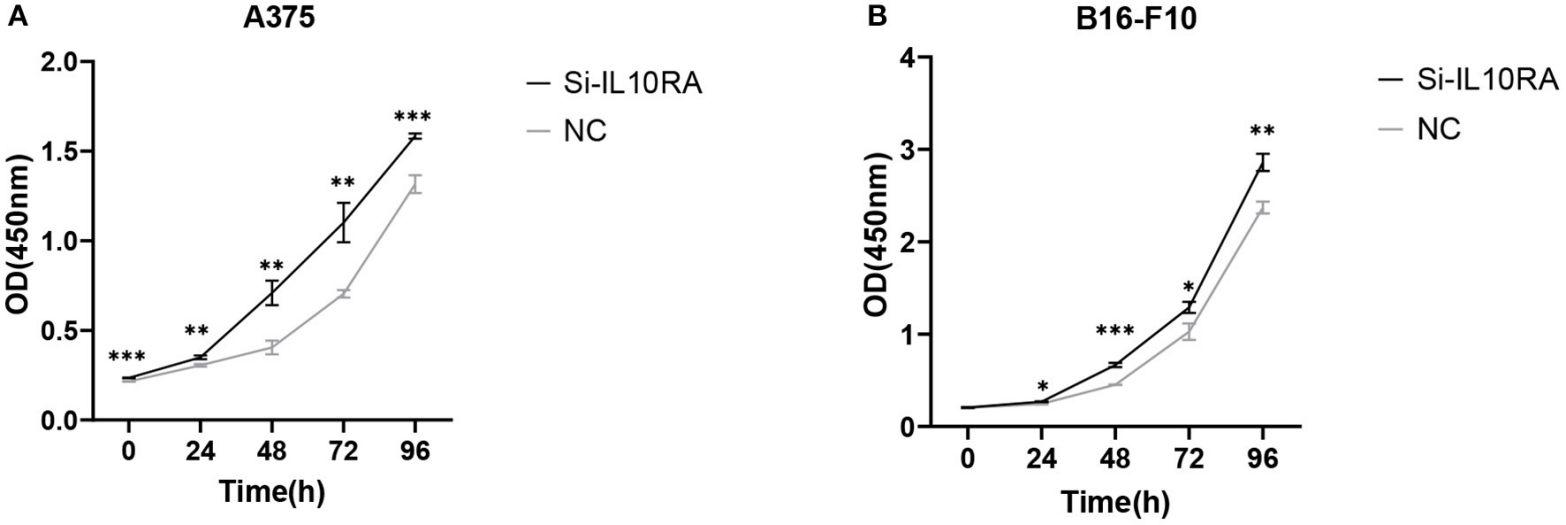
The effect of IL10RA on cell proliferation *in vitro* using CCK8 assays after downregulating IL10RA in A375 **(A)** and B16-F10 **(B)** cells. ^*^P < 0.05, ^**^P < 0.01, ^***^P < 0.001.

We further conducted *in-vitro* experiments to validate the function of IL10RA in inhibiting the proliferation, migration and invasion of melanoma cells. The CCK8 assays showed that melanoma cells with lower IL10RA expression exhibited stronger proliferation ability compared to that in control groups, as shown in Figure 7. For A375 cells, the intervention efficacy compared to control group at each time point is statistically significant. We observed the similar results on B16-F10 cells.

**Figure 8:**
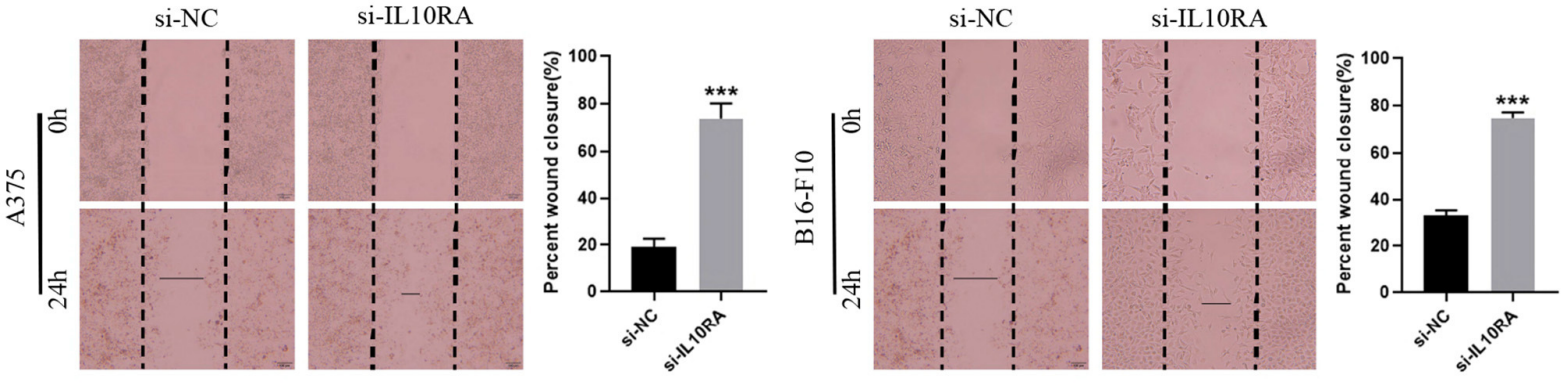
Cell migration abilities were assessed by wound-healing assays after downregulating IL10RA in A375 and B16-F10 cells. ^***^P < 0.001.

**Figure 9:**
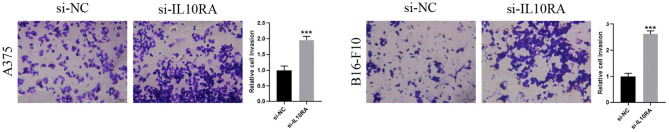
Cell invasion abilities were assessed by transwell assays after downregulating IL10RA in A375 and B16-F10 cells. ^***^P < 0.001.

We next tested whether IL10RA can affect the migration of melanoma cells. After 24 h, A375 and B16-F10 cells transfected with si-IL10RA migrated to the middle were much larger than the control group, as shown in Figure 8 (*p* < 0.001). Therefore, wound healing assays demonstrated that knockdown of IL10RA can promote the migration of melanoma cells compared to control groups. In addition, in order to prove the effect of IL10RA on cell invasion ability, we also conducted the transwell experiments. After incubated at 37°C for 24 h, the number of A375 and B16-F10 cells transfected with si-IL10RA from the upper chamber to the lower chamber was larger that of the control group, which indicates that invasion of melanoma cells are promoted by IL10RA downregulation, as shown in Figure 9 (*p* < 0.001).

### 3.6. IL10RA Potentially Mediates in Metastatic Melanoma via PI3K-AKT Pathway

**Figure 10:**
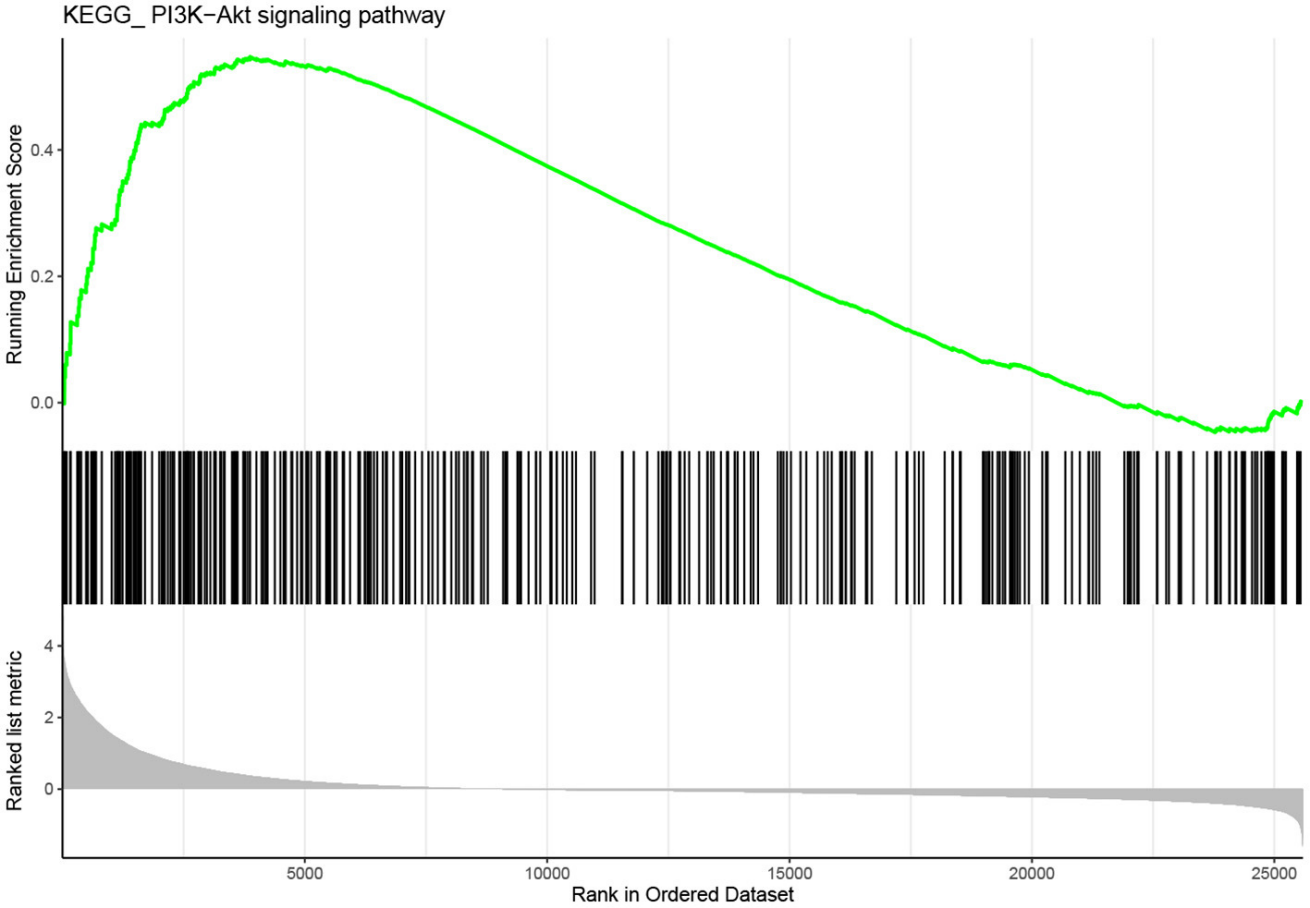
Gene set enrichment analysis (GSEA). The gene set of “PI3K-AKT signaling” was enriched in TCGA dataset with IL10RA highly expressed.

GSEA was conducted to explore further potential biological functions of IL10RA in metastatic melanoma. Based on the cut-off criteria, PI3K-AKT signaling pathway was enriched in metastatic melanoma samples with highly expressed IL10RA, as shown in Figure 10. Note that a multitude of researches have reported the role of PI3K-AKT in melanoma. For instance, activation of PI3K-AKT signaling appears to play a fatal role in brain metastases that stem from melanoma (Chen et al., 2014). Li et al. (2019) demonstrated that Ras-PI3K-AKT could promote cells proliferation and migration in uveal melanoma cells by downregulation of H3K56ac expression. Chang et al. (2020) found knockdown of the expression of AURKB could suppress cell growth and induced apoptosis in melanoma, which was mediated by inhibition of BRAF/MEK/ERKs and PI3K-AKT signaling pathways. Importantly, they also found that HI-511, a dual-target inhibitor against both AURKB and BRAF V600E, suppresses both drug-sensitive and -resistant melanoma development by inducing apoptosis and mediating the inhibition of the BRAF/MEK/ERKs and PI3K-AKT signaling pathways. It is reported that high expression of HSP90 and PI3K/AKT/mTOR pathway components in melanoma tumors and this expression correlates with poor survival in melanoma patients (Calero et al., 2017). These studies indicate that the activation of the PI3K-AKT signaling pathway has an significant correlation with the poor prognosis of melanoma.

It has been reported that IL-10 effectively immunoregulates the antigen-presenting cell (APC) function of dendritic cells (DCs) through suppression of the PI3K-AKT pathway (Bhattacharyya et al., 2004). Anti-inflammatory cytokine IL10 reduced S100A8 and 100A9 protein levels mediated via PI3K-AKT signaling in mononuclear cells of essential thrombocythemia (ET) (Diklić et al., 2020). PI3K/AKT signaling activation and EBV lytic induction responded to IL-10 knockdown (Gao et al., 2019). In addition, Mu et al. (2020) found that the PI3K agonist impaired the tolerance of Kupffer cells (KCs) to endotoxin and increased the phosphorylation levels of NF-κB in KCs, while the secretion of IL10 was significantly decreased. It can be concluded that IL10RA and PI3K-AKT pathway interact and influence each other. Extensive previous studies have shown that activation of PI3K-AKT pathway is associated with poor prognosis of melanoma. Therefore, we speculate that IL10RA may inhibit the metastasis of melanoma by regulating the PI3K-AKT signaling pathway, which we plan to do further exploration to verify the mediation of IL10RA in PI3K-AKT pathway.

## 4. Conclusion and Discussion

Melanoma accounts for a small proportion of all skin cancer cases but is the major cause of skin cancer-related deaths (Chuchu et al., 2018). In contrast to the steady or declining trends for other cancer types, the incidence of melanoma continues to rise up during the past 40 years (Siegel et al., 2014). Metastatic melanoma is the most aggressive form of skin cancer and advanced patients have limited treatment and poor prognosis, because transfer to distant sites and the internal organs is almost always incurable (Finn et al., 2012). Therefore, there is an urgent demand to explore the molecule mechanisms involved in the development and progression of metastatic melanoma. Weighted gene co-expression network analysis (WGCNA) has been proved to be an effective method to detect co-expressed modules and hub genes. In this study, we constructed a gene co-expression network based on WGCNA and identified 11 biomarker genes that were closely associated with prognosis. Among these biomarker genes, IL10RA shows highest correlation with clinically important module. Furthermore, our *in vitro* biochemical experiments verified that IL10RA is a strong biomarker for the prognosis of metastatic melanoma.

There are some reports regarding that IL10RA is not conducive to disease development. Aberrant IL10RA expression in melanoma creates autocrine circuitry involving endogenous IL10, which disrupts basal STAT3 phosphorylation and dampens the induction of anti-inflammatory signals, such as IL6, and slightly increases the resistance of A375 cells to apoptosis, thus showing that receptor absence is a critical mechanism for preventing this autocrine loop, enabling effective paracrine communication and controlling the cellular response (Kang et al., 2013). Béguelin et al. also found that gene expression of IL10, IL10RA, and IL10RB were remarkably overexpressed in diffuse large B-cell lymphomas (DLBCLs) which were dependent on IL10-STAT3 signaling and blocking the IL10R killed DLBCL cell lines through cell cycle arrest and induction of apoptosis due to the interruption of IL10-JAK-STAT signaling. Besides, they also demonstrated that the effect of IL10R inhibition derived from interruption of IL10-IL10R auto-stimulatory loop, for that anti-IL10R treatment led to the downregulation of IL10 secretion (Beguelin et al., 2015). Isabella et al. found higher levels of IL10RA is accompanied by a corresponding decrease in miR-15a, miR-185, and miR-211 in melanoma samples. IL10RA was a target of these miRNAs, and inhibition of them obviously promotes the proliferation in the melanoma cell lines, and this effect will be suppressed by specific knockdown of IL10RA (Venza et al., 2015). In this study, we demonstrated that high IL10RA expression is correlated with positive prognosis of metastatic malignant melanoma. In addition, our *in vitro* experiments proved that knockdown of IL10RA promotes the proliferation, migration and invasion of melanoma cells. Therefore, the role of IL10RA in metastatic malignant melanoma requires further verification in our future works.

## Data Availability Statement

The datasets generated for this study can be found in online repositories. The names of the repository/repositories and accession number(s) can be found in the article/Supplementary Material.

## Author Contributions

SC and ZL conceived the main idea and analyzed the data. ZS and ZF helped to improve the idea and the framework of the manuscript. SC and WZ drafted the manuscript. HL reviewed the drafts of the paper and improved the manuscript. SC collected the data and performed the statistical analysis. JL and HL supervised the study and provided funding. All authors read and commented on the manuscript.

## Conflict of Interest

The authors declare that the research was conducted in the absence of any commercial or financial relationships that could be construed as a potential conflict of interest.
