# Supplementary material for: Identification of IL10RA by Weighted Correlation Network Analysis and in vitro Validation of Its Association With Prognosis of Metastatic Melanoma
Source: Front Cell Dev Biol. 2021 Jan 8;8:630790. doi: 10.3389/fcell.2020.630790 (PMC7820192; doi:10.3389/fcell.2020.630790)
Supplement: Supplementary file 1 [file Data_Sheet_1.PDF]

Supplementary Material

1 FIGURES

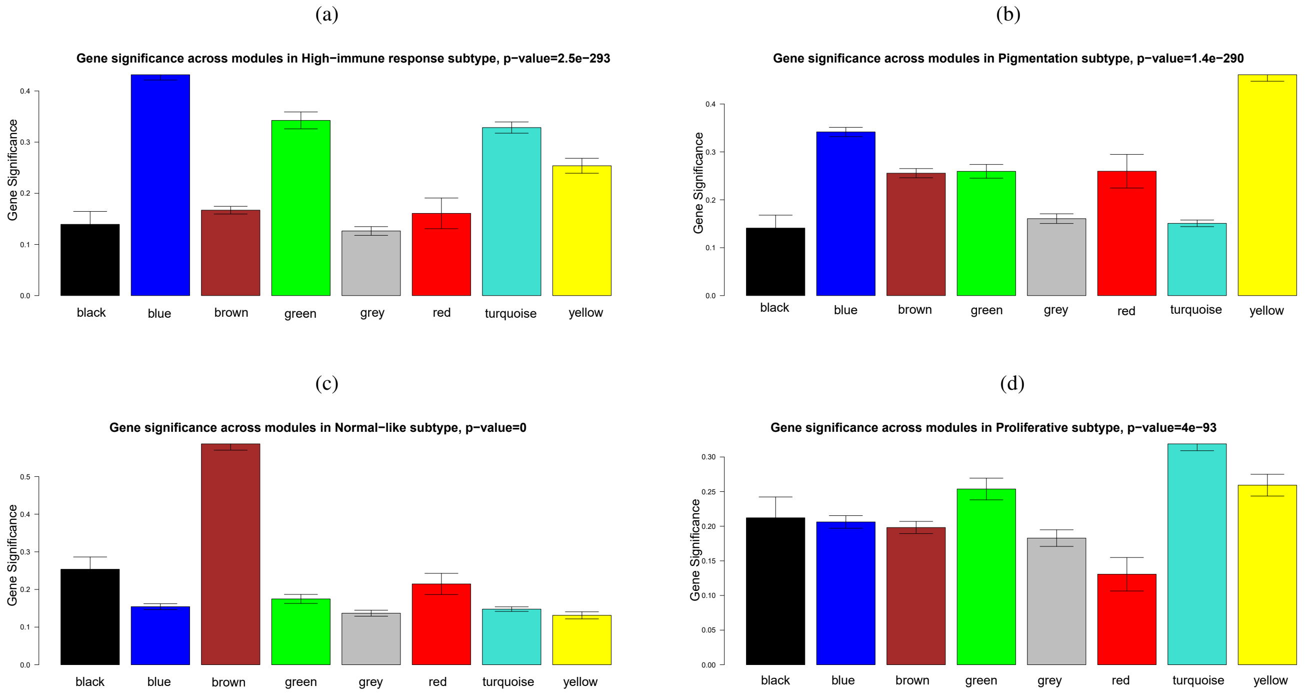

Figure S1: Distribution of average gene significance in the modules based on different subtypes. (a) Blue modules-highest association with High-immune response. (b) Yellow modules-association with Pigmentation. (c) Brown modules-highest association with Normal-like. (d) Turquoise modules-highest association with Proliferative.

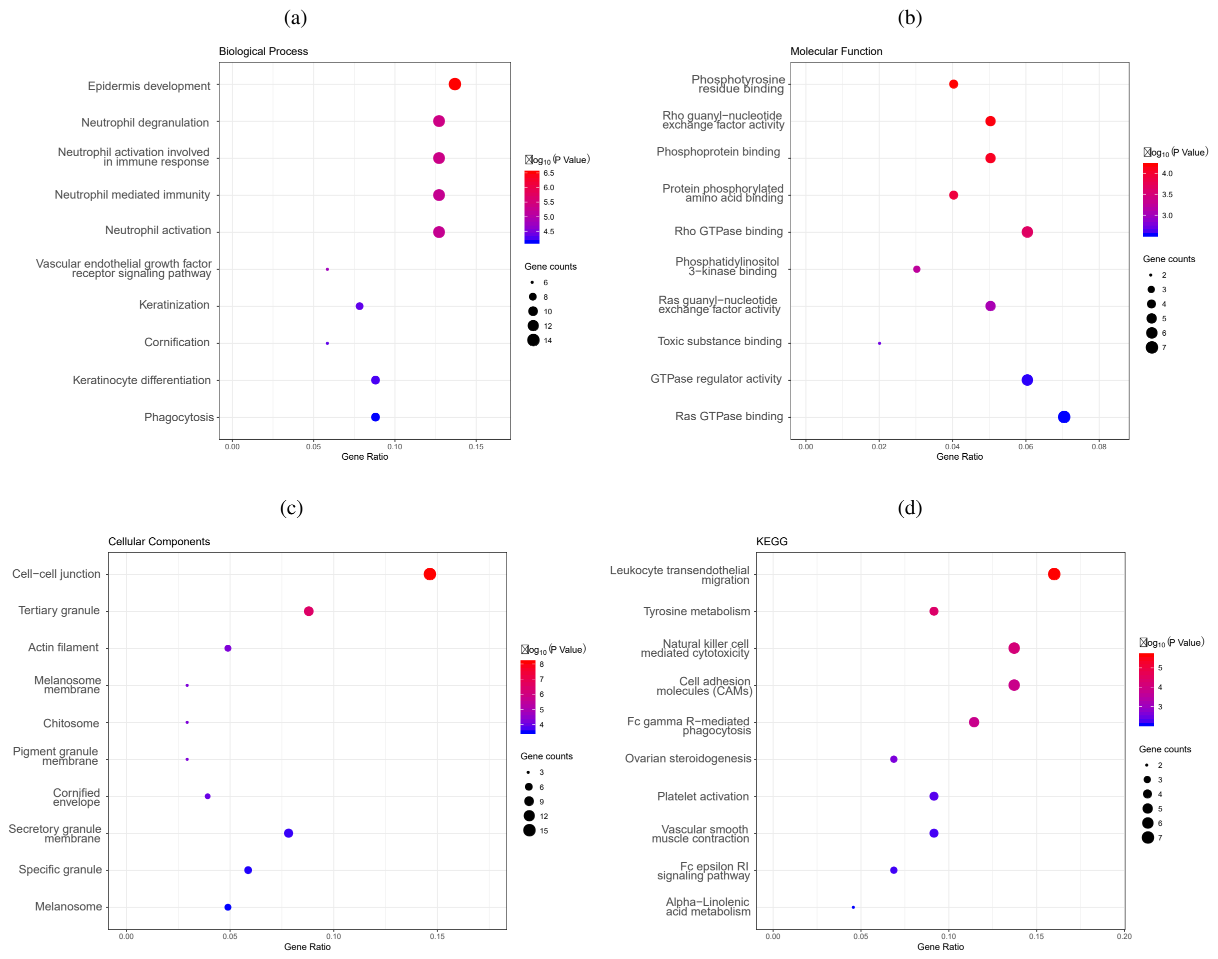

Figure S2: GO and KEGG pathway enrichment analysis. (a) Biological process analysis. (b) Molecular function analysis. (c) Cellular component analysis. (d) KEGG pathway analysis.

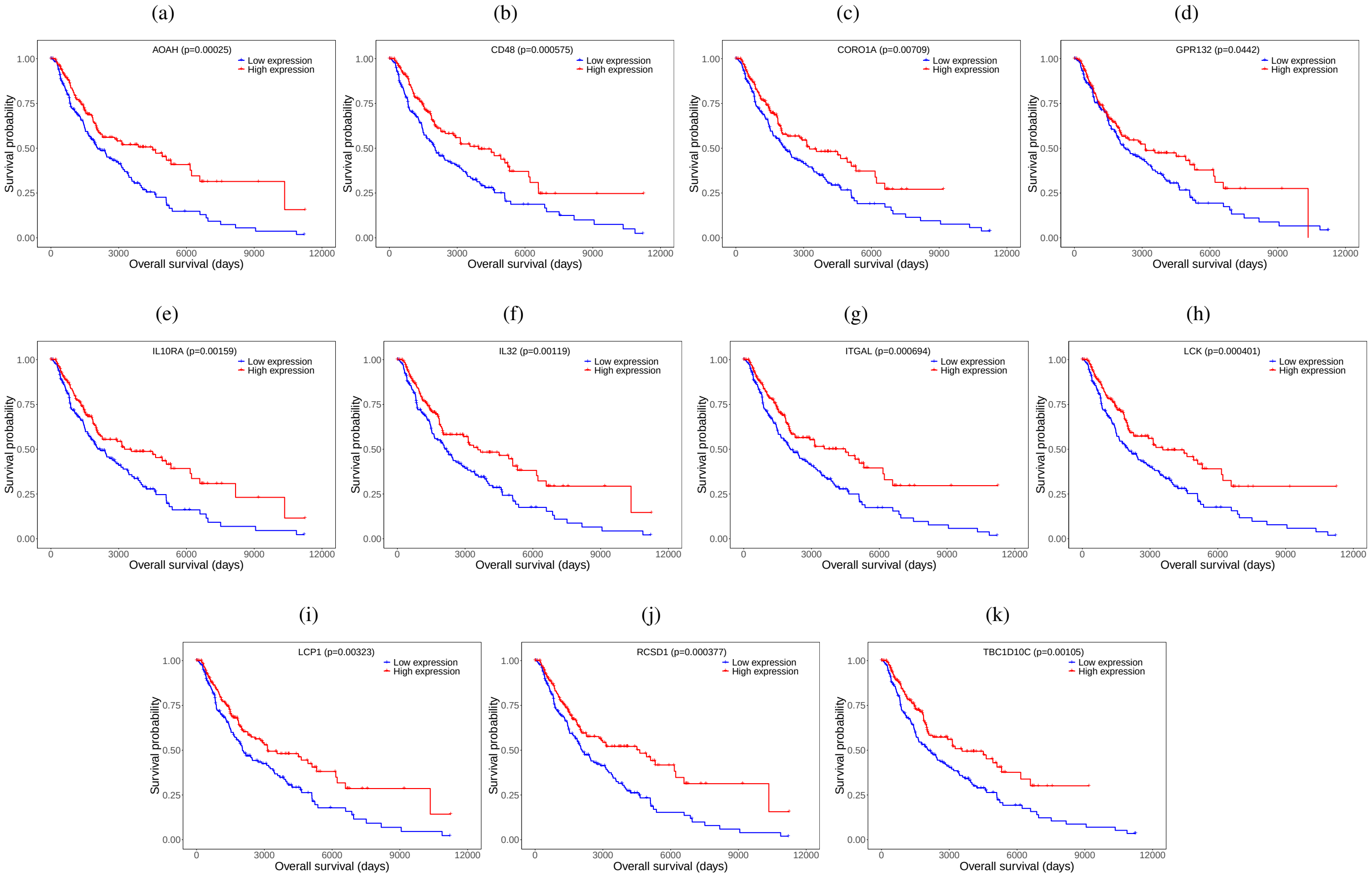

Figure S3: Survival analysis of 11 hub genes in metastatic melanoma in TCGA dataset (Red lines represent the samples with a highly expressed gene and the blue lines represent samples with a lowly expressed gene).
